# Mediastinal Lymphadenopathy, Class-Switched Auto-Antibodies and Myocardial Immune-Complexes During Heart Failure in Rodents and Humans

**DOI:** 10.3389/fcell.2020.00695

**Published:** 2020-08-07

**Authors:** Amalia Sintou, Catherine Mansfield, Alma Iacob, Rasheda A. Chowdhury, Salomon Narodden, Stephen M. Rothery, Robert Podovei, Jose L. Sanchez-Alonso, Elisa Ferraro, Pamela Swiatlowska, Sian E. Harding, Sanjay Prasad, Nadia Rosenthal, Julia Gorelik, Susanne Sattler

**Affiliations:** ^1^National Heart and Lung Institute, Imperial College London, London, United Kingdom; ^2^Royal Brompton Hospital, Royal Brompton and Harefield NHS Foundation Trust, London, United Kingdom; ^3^The Jackson Laboratory, Bar Harbor, ME, United States

**Keywords:** chronic heart failure, myocardial infarction, autoimmunity, auto-antibodies, anti-heart, adaptive immune system

## Abstract

Mediastinal lymphadenopathy and auto-antibodies are clinical phenomena during ischemic heart failure pointing to an autoimmune response against the heart. T and B cells have been convincingly demonstrated to be activated after myocardial infarction, a prerequisite for the generation of mature auto-antibodies. Yet, little is known about the immunoglobulin isotype repertoire thus pathological potential of anti-heart auto-antibodies during heart failure. We obtained human myocardial tissue from ischemic heart failure patients and induced experimental MI in rats. We found that anti-heart autoimmunity persists during heart failure. Rat mediastinal lymph nodes are enlarged and contain active secondary follicles with mature isotype-switched IgG2a B cells. Mature IgG2a auto-antibodies specific for cardiac antigens are present in rat heart failure serum, and IgG and complement C3 deposits are evident in heart failure tissue of both rats and human patients. Previously established myocardial inflammation, and the herein provided proof of B cell maturation in lymph nodes and myocardial deposition of mature auto-antibodies, provide all the hallmark signs of an established autoimmune response in chronic heart failure.

## Graphical Abstract

The adaptive anti-heart immune response in CHF. A myocardial infarction initiates an adaptive immune response with activation of the heart draining mediastinal lymph nodes, formation of secondary follicles and B cell maturation with production of mature self-reactive auto-antibodies which may subsequently induce cardiomyocyte dysfunction and death.

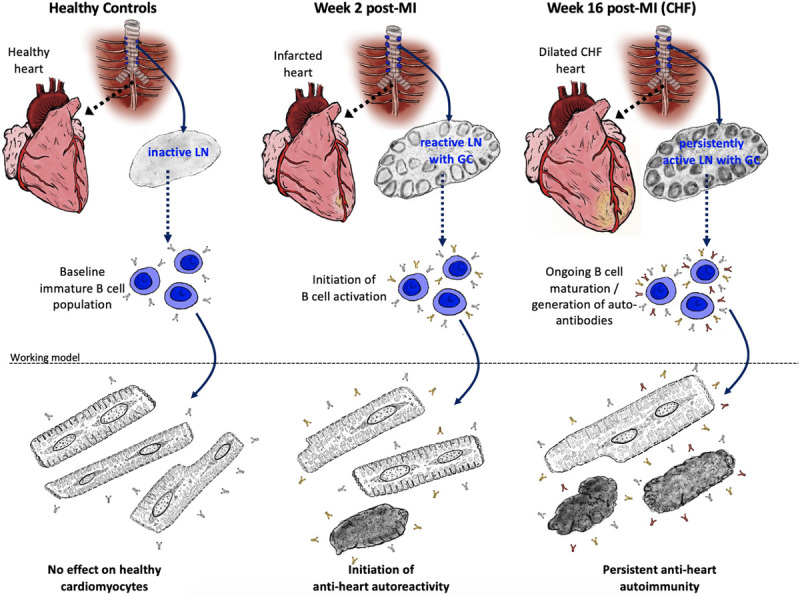

## Introduction

Chronic heart failure (CHF) describes a pathological condition in which the heart is unable to pump enough blood to meet the metabolic demands of the body. It is the final outcome of a range of disorders including ischemic heart disease following myocardial infarction (MI) (Torabi et al., 2008), and a major public health problem affecting 26 million people worldwide (Ziaeian and Fonarow, 2016).

The immune system has been implicated in the immediate response to MI, and is crucial for quick tissue repair (Nahrendorf and Swirski, 2016). However, the post-MI immune response does not cease once acute inflammation has been resolved, and excessive activation of endogenous repair mechanisms may lead to ongoing inflammation, fibrosis, and sustained tissue damage (Sattler et al., 2017). Cardiac self-antigens released from necrotic cardiomyocytes in a highly inflammatory environment also activate and trigger myocardial infiltration of autoreactive B and T cells of the adaptive immune system (Varda-Bloom et al., 2000; Yan et al., 2013; Zouggari et al., 2013; Tang et al., 2019). These can be long-lived and may cause sustained damage to the myocardium.

The presence of anti-cardiac auto-antibodies in post-MI serum is a well-known clinical phenomenon (Kaya et al., 2012). Post-MI Dressler syndrome was first described in the 50s and is now recognized as an adaptive immune response against myocardial proteins (Spodick, 2004). However, besides rare cases of clinically obvious extreme manifestations, persistent subclinical auto-reactivity can cause ongoing damage to previously healthy myocardial tissue, which may increase the risk of adverse remodeling and heart failure. Notably, enlargement of the mediastinal lymph nodes draining the heart has been observed during heart failure, but was explained by hemodynamic mechanisms related to cardiac decompensation (Ngom et al., 2001; Pastis et al., 2011). Activated B cells generate tissue-specific auto-antibodies and pro-inflammatory cytokines, which can directly contribute to cardiac dysfunction. Activated B and T cells interact in germinal centers of draining lymph nodes to facilitate B cell affinity maturation and class switching to mature IgG isotypes, which convey a range of effector functions (Hamel et al., 2012). Information on the auto-antibody isotypes in post-MI and CHF patients is limited, and further research may yield fundamental insights into pathological effects of auto-antibodies present in these patients.

In the present study, we document an active adaptive immune response against the heart with the presence of mature class-switched anti-heart auto-antibodies in the serum as well as deposited in the myocardium in both rats with experimental heart failure and human heart failure patients. We also detected myocardial deposition of complement, which suggests immune-complex mediated pathology.

## Materials and Methods

### MI Surgery

All animal procedures were approved by the Imperial College Governance Board for Animal Research and in accordance with the United Kingdom Home Office Animals (Scientific Procedures) Act 1986 and Directive 2010/63/EU of the European Parliament on the protection of animals used for scientific purposes. 30 Sprague Dawley (all male, 250 to 350 g) were obtained from Charles River Laboratories, United Kingdom. MI was induced by LAD ligation as described previously (Mawad et al., 2016). Anesthesia was induced with 5% isoflurane and maintained at 2% after intubation. Perioperative analgesic regime included buprenorphine (0.05 mg/kg) and Rimadyl (5 mg/kg), Baytril (5 mg/kg), and sub-cutaneous 1 ml of 0.9% saline for hydration. After left parasternal incision, thoracotomy through the fourth intercostal space and removal of the pericardium, ligation of the LAD was performed 1–2 mm distal to the inferior border of the left atrium using a 7–0 Prolene suture. The thoracotomy, chest wall and the skin were closed using 4–0 Vicryl sutures. Daily doses of Rimadyl were administered for at least 48 h after surgery for post-operative analgesia.

### Human Tissue Samples

All work was carried out under the guidelines of the Human Tissue Act 2004 and conform to the principles outlined in the Declaration of Helsinki. Only fully anonymized tissue and corresponding clinical data was available to researchers. Human left ventricular tissue sections were obtained from hearts of end-stage heart failure patients removed during transplant surgery (REC: 09/H0504/104+5; Biobank approval number: NP001-06-2015). Control samples were prepared from organ donor hearts assessed to be unsuitable for donation (REC: 16/LO/1568). Informed consent was obtained from each patient involved in this study or next of kin in case of donors. Patient characteristics: HF due to ischemic heart disease; 2 females (45, 60 years), 5 males (57–67 years); HF due to myocarditis; 2 females (20, 28 years), 1 male (51 years); donors; 3 females (17, 38, 50 years), 2 males (48, 39 years). CMR images were acquired on a 1.5 T scanner to exemplify mediastinal lymphadenopathy in the context of ischemic cardiomyopathy with a 2-month old transmural myocardial infarction in the circumflex artery territory and mild LV systolic dysfunction.

### Adult Rat Cardiomyocyte Isolation

Euthanasia was performed by cervical dislocation after induction of terminal anesthesia using 5% isoflurane. Hearts were excised, cannulated via the aorta and perfused with Krebs-Henseleit buffer (KH) at 37°C using a Langendorff apparatus. After blood was cleared from the coronary circulation, KH buffer was switched to a low calcium (LoCa2+) buffer to stop contraction. A solution of 1 mg/ml Collagenase II and 0.6 mg/ml Hyaluronidase (C+H) in enzyme buffer was perfused into the heart for 10 min after which it was minced in fresh C+H buffer. Heart samples were disrupted mechanically at 35°C for 5 min followed by further 30 min and the supernatant filtered through gauze. Filtrates were centrifuged for 1 min at 700 rpm and cardiomyocyte pellets re-suspended. Isolated primary adult cardiomyocytes were cultured in modified M199 medium (Thermo Fisher Scientific, Rochford, United Kingdom) and treated with post-MI sera at a dilution of 1:10 for 24 and 48 h.

### qPCR

Cells were taken up in TRIzolTM (both Gibco, Thermo Fisher Scientific, Dartford, United Kingdom) and RNA isolation was performed using phenol-chloroform extraction. RNA concentration and purity were determined by using a NanoDrop spectrophotometer (ND-8000-GL, Thermo Fisher Scientific, Rochford, United Kingdom). RNA cleanup was performed using the RNeasy Mini Kit (Qiagen, Hilden, Germany) according to manufacturer’s instructions and RNA concentration was adjusted to 500 ng/μl. cDNA was synthesized using the RT2 first strand kit. qPCR was performed using RT2 SYBR^®^ Green qPCR Mastermix and the Rat T-Cell and B-Cell Activation RT2 Profiler qPCR array (all Qiagen, Hilden, Germany), according to manufacturer’s protocols using a RealPlex2 Eppendorf Mastercycler^®^. Threshold cycle (*C*_*t*_) values of the target genes were normalized to the experimental control. Data analysis was performed using the 2−ΔΔCt method.

### Immunofluorescence Staining

For analysis of auto-antibody binding, healthy rat hearts were frozen in OCT (Sigma-Aldrich, Gillingham, United Kingdom) and cut into 5 μm sections. Control, 2- and 16-weeks post-MI sera were used as primary antibodies in 1:100 dilution and incubated overnight at 4°C. Sections were washed 3 times and a secondary anti-rat IgG AlexaFluor^®^ 488 antibody (BioLegend, London, United Kingdom) was used for detection. Wheat germ agglutinin (WGA)-AlexaFluor^®^ 594 (Invitrogen, Thermo Fisher Scientific, Rochford, United Kingdom) was used as membrane counterstain. Sections were mounted with DAPI-containing mounting medium (Vectashield, Vector Laboratories, Peterborough, United Kingdom). For detection of IgG2a+ cells in mediastinal lymph nodes, frozen LN sections were stained with Alexa Fluor^®^ 488 Goat anti-rat IgG and Alexa Fluor^®^ 647 anti-rat IgM (both BioLegend, London, United Kingdom). IgG and complement C3 deposits in rat and human CHF tissue were detected using; Alexa Fluor^®^ 488 goat anti-rat IgG and rabbit anti-rat C3 with Alexa Fluor^®^ 488 goat anti-rat IgG (all BioLegend, London, United Kingdom), rat anti-human IgG with Alexa Fluor^®^ 488-anti-rat IgG and mouse anti-human C3 (Abcam, Cambridge, United Kingdom) with Alexa Fluor^®^ 488 Goat anti-mouse IgG. Sections were mounted using DAPI mounting medium (Vectashield, Vector Laboratories, Peterborough, United Kingdom). Mean fluorescent intensity (MFI) of IgG and C3 deposition was quantified using FIJI/ImageJ (Schneider et al., 2012). Obtained MFI of fully stained sections were normalized against corresponding control sections of the same animal to correct for variations in cardiomyocyte autofluorescence.

### ELISA

ELISA plates (SpectraMax Paradigm Molecular-Devices, United Kingdom) were coated with 50 μl per well of 4 μg/μl pig full heart lysate (Novus Biologicals, Bio-Techne, Abingdon, United Kingdom) recombinant pig myosin (Sigma-Aldrich, Gillingham, United Kingdom) or mouse troponin I (Abcam, Cambridge, United Kingdom) diluted in PBS overnight at 4°C. Plates were washed 3 times for 5 min each with 200 μl per well of ELISA washing buffer. Then, 50 μl of rat serum per well was added in either 1:10 or 1:100 dilutions for overnight incubation at 4°C. Detection reagents were anti-rat IgG-HRP or IgG1/2a/2b/2c-Biotin combined with Streptavidin-HRP (all BioLegend, London, United Kingdom).

For relative quantification of antibody isotypes in the serum, a Rapid Antibody Isotyping Assay Kit, rat (Thermo Fisher Scientific, Rochford, United Kingdom) was used according to the manufacturer’s instructions using previously determined serum dilutions (Hasham et al., 2017). For all ELISA assays, detection steps were performed using a TMB ELISA buffer kit (Peprotech, London, United Kingdom) as per manufacturer’s instructions. An ELISA plate reader (SpectraMax Paradigm Molecular-Devices, United Kingdom) was used to measure light absorbance at 450 nm for the HRP product and 570 nm for the plastic background.

### Statistical Analysis

Statistical analysis was performed using SPSS or GraphPad Prism 6 and data were presented as mean ± SEM. 2-group comparisons were analyzed using 1- or 2-tailed unpaired Student’s *t*-tests. Comparison between multiple groups was performed using ANOVA with Dunnett’s multiple comparisons post hoc test to obtain multiplicity-adjusted *p*-values. Datasets of *n* > 10 that failed the D’Agostino and Pearson omnibus normality test were analyzed using non-parametric Kruskal-Wallis test with Dunn’s multiple comparisons post hoc test to obtain multiplicity-adjusted *p*-values. Differences were considered significant at *p* < 0.05.

## Results

### Persistent Reactivity of Heart-Draining Lymph Nodes During CHF

**FIGURE 1:**
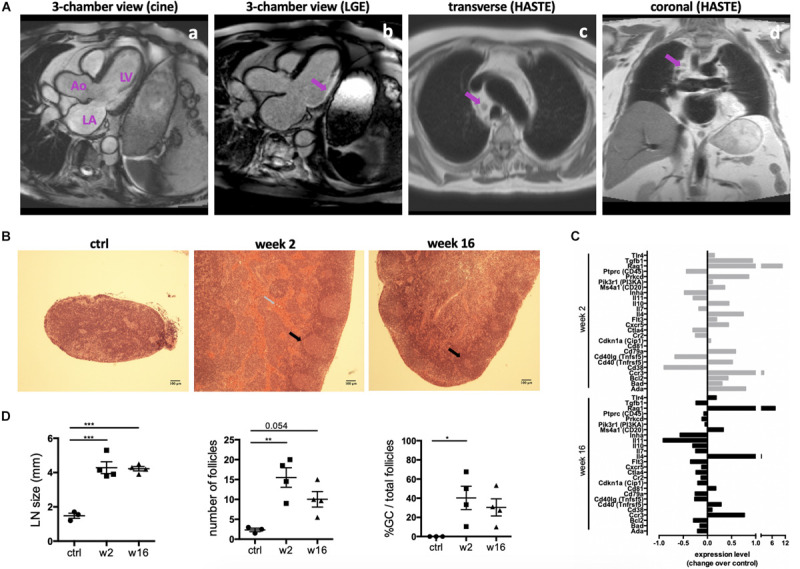
Persistent activation of heart-draining lymph nodes at CHF stage after infarction. **(A)** Example of 1.5 T cardiac MRI images of mediastinal lymphadenopathy in a patient with ischemic cardiomyopathy 2 months after transmural myocardial infarction in the circumflex artery territory. (a) 3-chamber cine view of the heart before gadolinium injection showing the left ventricle (LV), left atrium (LA) and aorta (Ao). (b) 3-chamber view of the heart showing late gadolinium enhancement (LGE) and transmural myocardial infarction in the infero-lateral wall of the LV. (c,d) Cardiac MRI transverse (c) and coronal (d) HASTE sequences showing mediastinal lymphadenopathy (arrows) in a patient with ischemic cardiomyopathy. **(B–D)** Myocardial infarction was induced by surgical ligation of the LAD. Mediastinal (heart draining) lymph nodes were obtained from healthy rats (ctrl), 2 weeks (acute post-MI) and 16 weeks (CHF stage) post-MI. **(B)** Histology (H&E) sections of representative lymph nodes. Post-MI rats show distinct lymphoid follicles containing active germinal centers (black arrows). Hemorrhage (gray arrow) is present in the medullary sinuses of the week 2 post-MI lymph nodes, indicating that they drain an area of hemorrhage. **(C)** Lymph node size and quantification of the total number of follicles and the percent of follicles with germinal centers. Data are expressed as mean +/–SEM for *n* = 3 (ctrl) and 4 (week 2, 16) animals, *p < 0.05, **p < 0.005, ***p < 0.001 (ANOVA with Dunnett’s multiple comparisons post hoc test). **(D)** qPCR Array testing a range of genes involved in B cell activation. Bars represent expression change over baseline, with healthy baseline values set to 0. Experiment performed in triplicates on 3 pooled spleens of ctrl (healthy baseline), week 2 and week 16 post-MI rats.

An MI is a potent trigger for an acute innate immune reaction, including early infiltration of neutrophils and macrophages (Nahrendorf and Swirski, 2016). Evidence is accumulating that T and B cells are also activated within a week post-MI (Kaya et al., 2012). Importantly, as described previously (Slanetz et al., 1998; Pastis et al., 2011), mediastinal lymphadenopathy is a common finding in patients with heart failure due to ischemic heart disease (Figure 1A). To further investigate if this phenomenon is indicative of the activation of the adaptive immune system in CHF, myocardial infarction was induced in male Sprague Dawley rats by surgical ligation of the left anterior descending (LAD) coronary artery. Heart-draining mediastinal lymph nodes were isolated at week 2 and week 16 after infarct surgery and analyzed for signs of reactivity. They showed distinct lymphoid follicles containing active germinal centers (GC) acutely post-MI (2 weeks) as well as at chronic stage (16 weeks) post-MI. Blood was visible in the medullary sinuses of the week 2 post-MI lymph nodes, indicating that they drained an area of hemorrhage (Figure 1B), which is in line with the myocardial damage induced by LAD. Quantification of lymph node size, total number of follicles and the percentage of follicles with GC confirmed an activated state at week 16, albeit decreased compared to week 2 (Figure 1C). Transcription of genes involved in B cell development and activation, including *Rag1*, *Cd20(MS4a1)*, *Cd81* was upregulated in the spleen 2 weeks post-MI, and some were still elevated over baseline at CHF stage (Figure 1D). Recombination activating gene (*Rag1*) was elevated most prominently in both week 2 and week 16, consistent with its role in B cell development and Ig formation. *Rag* expression is also upregulated in antigen-activated early memory B cells during autoimmune responses (Wang and Diamond, 2008). Sustained upregulation of *Il4* and the Th2-associated chemokine receptor *Ccr3* together with an increase in *Cd40* (*Tnfrsf5*) but a downregulation of *Cd40lg* (*Tnfsf5*) may provide an environment blocking apoptotic cell death and inducing sustained B cell growth and differentiation (Nakanishi et al., 1996; Toellner, 2014).

### The CHF Antibody Repertoire Shifts Toward Mature Class-Switched Isotypes

**FIGURE 2:**
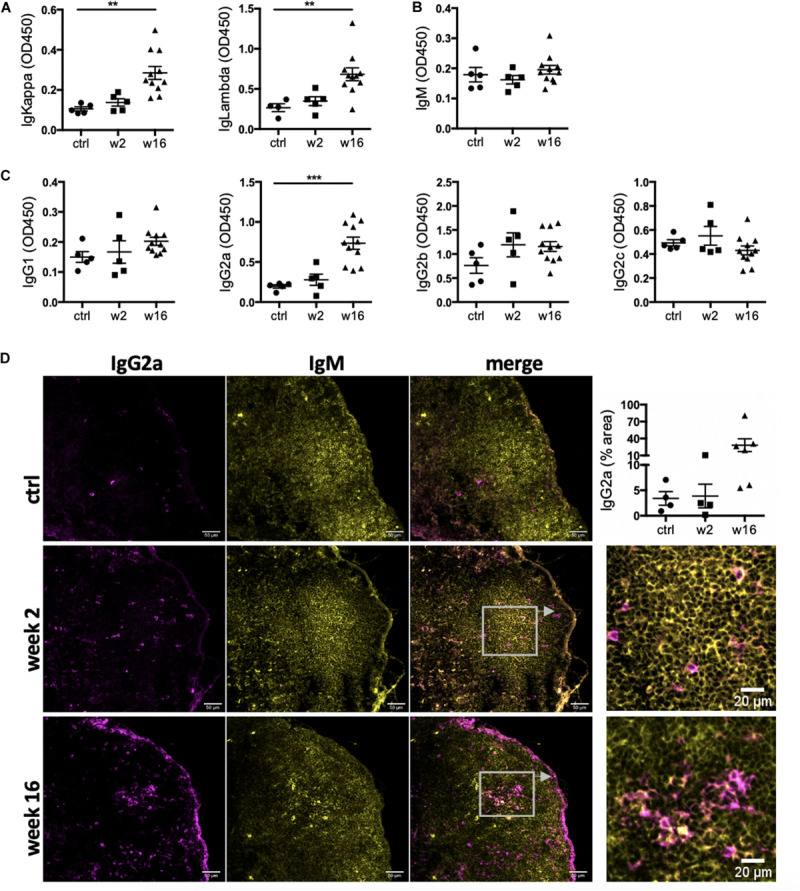
Levels of IgG2a antibodies in the circulation and IgG2a+ cells in the heart-draining lymph nodes are elevated during CHF. Myocardial infarction was induced by surgical ligation of the LAD. Serum was collected 2 weeks (acute post-MI) and 16 weeks (chronic heart failure stage) post-MI and analyzed by ELISA for relative concentration of **(A)** immunoglobulin (Ig) light chains (IgKappa and IgLambda) and **(B,C)** heavy chains (immature IgM **(B)**, and mature class-switched IgG1, IgG2a, b and c **(C)**. n = 5 (ctrl, week 2) and 11 (week 16) / group **(D)** Immunofluorescence staining of mediastinal lymph nodes with anti-rat IgM (green) and anti-rat IgG2a (red). *n* = 4 (ctrl, week 2) and 6 (week 16) / group, Data are expressed as mean +/–SEM, **p < 0.001, ***p < 0.0001 (ANOVA with Dunnett’s multiple comparisons post hoc test).

The presence of active GC in heart-draining lymph nodes during CHF marks an ongoing B cell maturation process. To assess maturity of the CHF antibody repertoire, we characterized the isotype composition of the local and systemic antibody repertoire in response to MI and their potential for pathological effects. Serum was collected 2 and 16 weeks post-MI and analyzed by ELISA for relative concentrations of immunoglobulin (Ig) light chains (IgKappa and IgLambda) and heavy chains (immature IgM, mature class-switched IgG1, IgG2a–c). A dramatic increase in overall antibody levels was evident (Figure 2A). IgM levels were comparable to baseline levels at both week 2 and week 16 (Figure 2B), likely due to baseline levels of natural IgM (Haas et al., 2010) or a missed early peak. Mature IgG isotypes on the other hand were increased, most prominently IgG2a (Figure 2C). This was accompanied by a marked increase of IgG2a^+^ cells in the mediastinal lymph nodes, indicating heart specificity of mature class-switched autoreactive B cells at CHF stage (Figure 2D). As expected for mature class-switched B cells (Zhang et al., 2013), IgG2a+ cells were detected within and around the GC. Another striking accumulation was observed in the subcapsular sinuses, where mature B cells have previously been shown to interact with antigen (Carrasco and Batista, 2007; Phan et al., 2007) thus putting them in the correct position to quickly react to cardiac antigen drained from the heart.

### CHF Serum Contains Mature Anti-heart Auto-Reactive Antibodies

**FIGURE 3:**
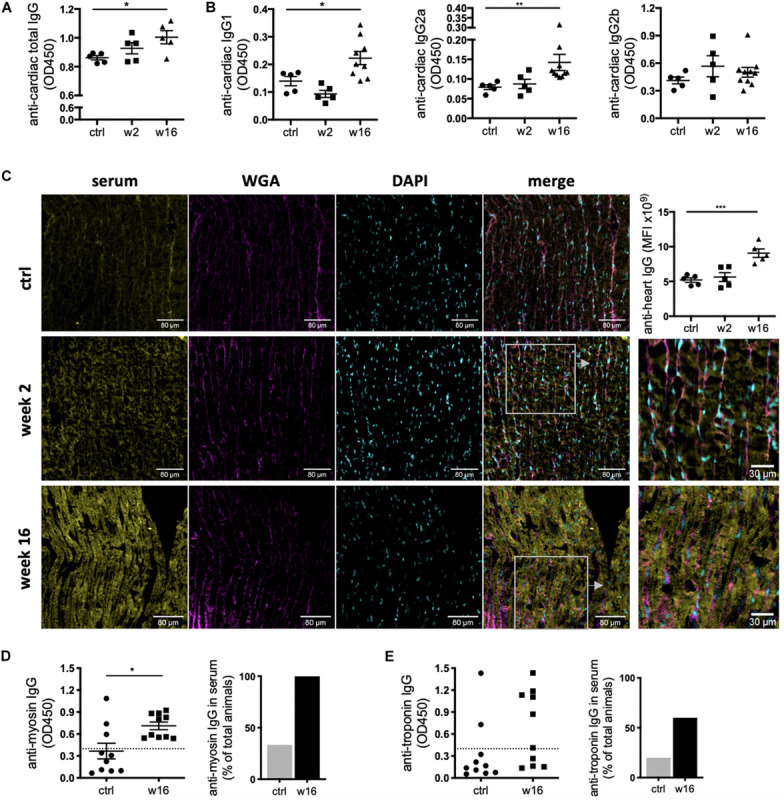
CHF serum contains anti-heart auto-reactive antibodies of mature class-switched isotypes. Myocardial infarction was induced by surgical ligation of the LAD. Serum was collected 2 weeks (acute post-MI) and 16 weeks (chronic heart failure stage) post-MI and analyzed by ELISA and immunofluorescence microscopy for the presence of heart specific auto-antibodies. **(A,B)** ELISA using post-MI serum against rat heart lysate (protein fraction) showing a progressive increase in the levels of **(A)** total IgG (*n* = 5 / group) and **(B)** IgG1, IgG2a, and IgG2b isotype auto-antibodies reactive against the heart (*n* = 5 (ctrl, week 2) and 10 (week 16) / group). **(C)** Representative images and quantification of immunofluorescence staining using post-MI serum on frozen sections of healthy rat hearts to assess binding of auto-antibodies to cardiac structures. Staining intensity was quantified by measuring mean fluorescence intensity (MFI) using FIJI/ImageJ. **(D,E)** ELISA using post-MI serum against recombinant pig myosin **(D)** and mouse troponin I **(E)**. Dotted line represents threshold for calculation of % of positive animals. Data are expressed as mean +/–SEM, *p < 0.05, **p < 0.001, ***p < 0.0001 [ANOVA with Dunnett’s multiple comparisons post hoc test **(A–C)**, one-tailed Student’s *t*-test with Welch correction **(D)**].

While the degree of overall increase in total antibody concentration and elevation of potentially pathological isotypes such as IgG2a in the periphery and the draining lymph nodes is striking in itself, CHF serum also contained auto-antibodies specific to cardiac protein. An ELISA assay using post-MI serum against coated healthy heart lysate showed a progressive increase in the levels of anti-heart IgG auto-antibodies (Figure 3A). Characterization of the heart-specific auto-antibody population confirmed that it also contains mature class-switched IgG1, IgG2a, and IgG2b isotype antibodies (Figure 3B). Although individuals show variable levels of IgG2a and IgG2b anti-heart auto-antibodies at CHF stage, elevation over baseline is evident in all animals.

A variety of major cardiac proteins, including cardiac myosin and troponin I, are targeted by auto-antibodies post MI (Kaya et al., 2012). To assess binding of auto-antibodies to cardiac structures, CHF and control sera were used to stain frozen sections of healthy rat hearts. Strong staining was observed when using CHF serum, with a pattern resembling cardiomyocyte striations. This confirms auto-antibodies specific to the cardiomyocyte contractile apparatus are present in CHF serum (Figure 3C). ELISA assay against recombinant myosin and troponin I confirm the presence of mature IgG auto-antibodies against the cardiomyocyte contractile machinery in rat CHF serum (Figures 3D,E). Notably, while all CHF rats harbor anti-myosin antibodies, anti-troponin antibodies are only found in 60% of CHF animals, indicating stronger immunogenicity of myosin in the post-MI process of autoimmune induction.

### Immunoglobulin G and Complement C3 Deposit in Myocardial Tissue During CHF

**FIGURE 4:**
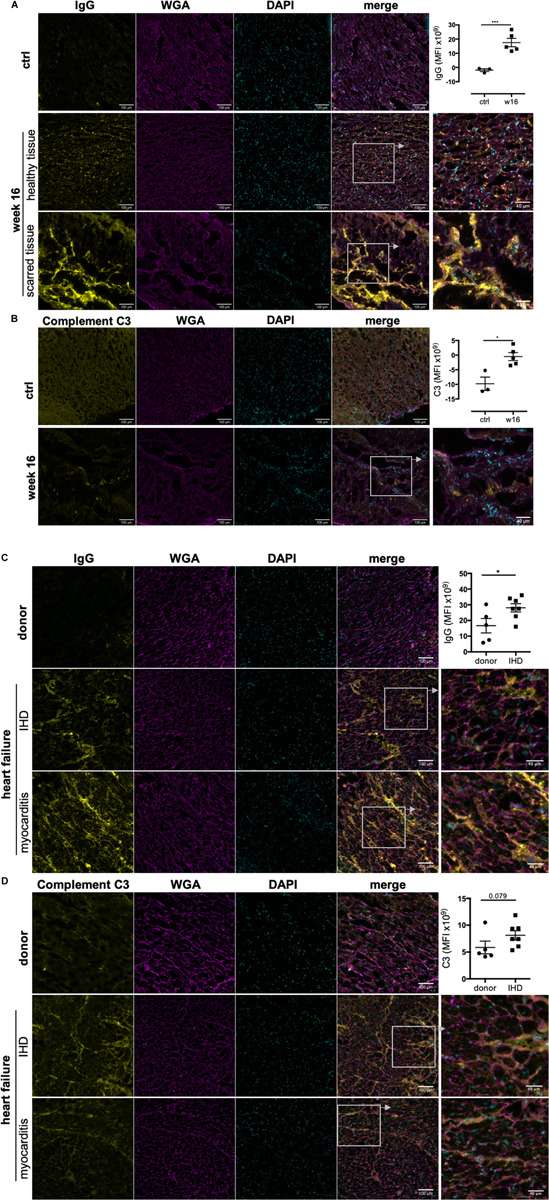
Immunoglobulin G and complement C3 deposit in rodent and human CHF heart tissue. MI was induced by surgical ligation of the LAD and heart tissue was collected during CHF after 16 weeks of MI. Human left ventricular tissue sections were obtained from end-stage heart failure patients during transplant surgery. Control samples for comparison were obtained from organ donor hearts assessed to be unsuitable for donation. Representative images and corresponding quantification (mean fluorescence intensity) of immunofluorescence staining against IgG and complement C3 on frozen sections of CHF rat hearts **(A,B)** and end-stage human HF heart **(C,D)** to assess *in vivo* IgG and C3 deposition on cardiac structures, respectively. rat *n* = 3 (control), 5 (CHF); human *n* = 5 (donors), 7 (CHF), 3 (myocarditis). Staining intensity was quantified by measuring mean fluorescence intensity (MFI) using FIJI/ImageJ. Data are expressed as mean +/–SEM, *p < 0.05, ***p < 0.001 (one-tailed Student’s *t*-test with Welch correction).

The ability to reach and bind to antigenic epitopes *in vivo* is crucial for the pathological function of auto-antibodies. Importantly, IgG deposition was detected in frozen heart tissue obtained from CHF rats, confirming anti-heart auto-antibodies can reach their cardiac antigen and deposit within the tissue *in vivo*. Besides a significant amount of IgG in severely damaged and scarred tissue areas, we also observed deposition in apparently healthy areas of rat CHF hearts (Figure 4A). Immune-complex formation between autoreactive IgG and complement C3 and subsequent cytotoxicity is a prominent pathological mechanism in auto-immune-mediated tissue destruction. In line with this, we also detected complement C3 in rat CHF tissue (Figure 4B).

Notably, IgG and complement C3 were also detected in human end-stage HF tissue (Figures 4C,D). Left ventricular tissue was obtained from end-stage HF patients during transplant surgery, and control samples were obtained from organ donor hearts deemed unsuitable for transplantation. Donors were 3 female and 2 male individuals aged 17 to 50 years. HF patients were 2 female (45 and 60 years) and 5 male (57–67 years) individuals with end stage HF due to ischemic heart disease (IHD) after MI. We also included 3 patients (2 females of 20 and 28 years and 1 male of 38) with heart failure due to prior myocarditis as positive controls for confirmation of the presence of IgG and C3 in heart disease with established immunological etiology. We found IgG deposition in all besides 1 IHD HF samples (86%) compared to only 1 of 5 donor samples (20%) (Figure 4C). Complement C3 followed the same pattern although staining intensity was overall lower (Figure 4D). Both IgG and C3 were found in all myocarditis HF samples. Notably, the pattern of IgG and C3 deposition was consistent between IHD and myocarditis HF tissue. While strongest staining was observed in perivascular areas, interstitial staining between cardiomyocytes and along cardiomyocyte membranes was also evident.

The above confirms the ability of anti-heart auto-antibodies during late stages of heart failure to cause ongoing tissue injury, not only in already damaged tissue but also in apparently healthy areas of the heart in both rodents and humans.

## Discussion

Heart failure affects 26 million people worldwide (Ziaeian and Fonarow, 2016) and myocardial inflammation plays an important role in local tissue destruction (Frangogiannis, 2012). In addition to local inflammation, the presence of auto-antibodies against major cardiac proteins (Kaya et al., 2012) as well as mediastinal lymphadenopathy are recognized clinical findings in patients with heart failure (Slanetz et al., 1998; Joffre et al., 2004; Pastis et al., 2011). Importantly, all of these are hallmark signs of other prototype autoimmune diseases including rheumatoid arthritis (Bouta et al., 2015) type 1 diabetes (Leeth et al., 2016) and multiple sclerosis (Pröbstel et al., 2015). In addition, the role of the cardiac lymphatics in ischemic heart disease and heart failure has recently recieved attention (Henri et al., 2016; Brakenhielm and Alitalo, 2019). Yet while we are starting to appreciate that anti-heart autoantibodies as well as cardiac lymphatics may have a role in heart failure, mediastinal lymphadenopathy remains unexplored. The reasons behind this phenomenon are speculative and have previously been explained by hemodynamic mechanisms related to cardiac decompensation and increased generalized edema in the lungs also affecting the lymph nodes (Ngom et al., 2001; Joffre et al., 2004; Pastis et al., 2011).

In this study, we document an active autoimmune state against the heart during CHF using a rat heart failure model (Graphical Abstract). Rodent mediastinal lymph nodes contain a high number of IgG2a positive cells, representing mature B cells that class-switched to IgG2a. Importantly, *in vivo* deposition of IgG auto-antibodies and complement C3 in CHF heart tissue in both rodents and human patients confirms that these auto-antibodies reach cardiac tissue *in vivo* and will be able to damage previously unaffected areas of the myocardium. Upon activation, immature B cells undergo either clonal expansion for immediate antibody production or germinal center reactions in the lymph nodes. The germinal center reaction including somatic hyper-mutation and class-switch recombination creates high affinity antibodies with variable Fc portions (Hamel et al., 2012). The Fc portion of antibodies is of particular importance as it conveys their effector functions, which include (a) direct induction of cell death by receptor cross-linkage or blockade of receptor-ligand interactions, (b) recruitment of effector cells for antibody-dependent cell-mediated cytotoxicity (ADCC), or (c) antibody-dependent cellular phagocytosis (ADCP) by engagement of activating Fcγ receptors (FcγR) or complement-dependent cytotoxicity (CDC) (Lu et al., 2018). Depending on specificity and isotype, anti-cardiac auto-antibodies in CHF can therefore result in cardiac injury in a variety of ways (Li et al., 2006; Ludwig et al., 2017). Most notably, antibodies of IgG2 isotype including IgG2a and IgG2b play well established pathogenic roles in autoimmune diseases such as systemic lupus erythematosus (SLE) (Ehlers et al., 2006). As we reported previously in a rodent SLE model, anti-cardiac antibodies of IgG2a and IgG2b isotype are present in circulation and deposit in the hearts (Hasham et al., 2017). IgG auto-antibodies activate the complement cascade, which culminates in formation of the cytolytic membrane attack complex (MAC) comprising of complement C5b-9. The MAC forms a trans-membrane pore leading to necrotic death of the target cell (Bohana-Kashtan et al., 2004). At sub-lytic concentrations, MAC can induce caspase activation and apoptosis (Nauta et al., 2002). Complement is accepted as a mediator of additional damage during acute MI and after reperfusion (Yasojima et al., 1998; Griselli et al., 1999), and sub-lytic activity has been implicated in the development of dilated cardiomyopathy. C5b-9 correlates with myocardial immunoglobulin deposition and expression of TNF-α. *In vitro*, C5b-9 attack on cardiomyocytes induces nuclear factor (NF)-κB activation as well as transcription, synthesis, and secretion of TNF-α by the cardiomyocytes themselves (Zwaka et al., 2002). NF-κB activation and TNF-α both induce cardiomyocyte hypertrophy (Sun et al., 2007; Hamid et al., 2011) *in vitro*, and the presence of immune cells able to respond to auto-antibody deposition *in vivo* will further increase the number of pathological pathways that contribute to CHF.

In summary, we show that long after the initially ischemic trigger event, CHF serum still contains active auto-antibodies against the heart with potent pathological functional isotypes. This confirms that post-MI immune auto-reactivity is indeed likely to be a crucial contributing factor to adverse remodeling in remote areas of the myocardium. This offers new avenues for therapeutic intervention in heart failure. A large number of clinical trials have attempted to improve post-MI outcome by early immunomodulation, with a strong focus on acute inflammation and short term readouts (Panahi et al., 2018; Ridker et al., 2018). However, immunomodulatory strategies targeting the adaptive immune system with the aim to restore immunological tolerance to the heart may have more potent long-term benefits. Importantly, identification of CHF as another member of the expanding family of immune-mediated diseases thus opens the door for new experimental, diagnostic and therapeutic approaches.

### Study Limitations

In this study, we interpreted the presence of mature class-switched auto-antibodies against cardiac antigens as representative of anti-heart autoimmunity. We did not quantify the numbers of infiltrating B and T cells as this provides little information regarding a potential pathological role. In addition, very convincing prior data on post-MI B and T cell numbers exist for both mouse and humans (Yan et al., 2013; Zouggari et al., 2013; Saxena et al., 2014; Rieckmann et al., 2019; Tang et al., 2019) and it is anticipated that the rat follows a comparable trajectory of post-MI myocardial infiltration.

Cellular assays to characterize the phenotype and function of heart-infiltrating B and T cell populations will be informative. These are currently restricted by the lack of flow cytometry antibody reagents specific to rat B and T cell activation markers but may be possible in the near future. The current study provides direct evidence of B cell activation as shown by the existence of secondary follicles in the mediastinal lymph nodes, activation markers in qPCR, and most importantly IgG2 staining in the lymph nodes. Also, presence of IgG autoantibodies requires the full cascade of T cell-B cell activation upstream.

IgG and Complement C3 deposition in the myocardium indicate immunocomplex-mediated tissue pathology. However, additional mechanistic studies are needed to prove a direct pathological effect of anti-heart autoantibodies in CHF.

## Data Availability Statement

All datasets presented in this study are included in the article.

## Ethics Statement

The studies involving human participants were reviewed and approved by REC: 09/H0504/104+5; Biobank approval number: NP001-06-2015 REC: 16/LO/1568. The patients/participants provided their written informed consent to participate in this study. The animal study was reviewed and approved by the Imperial College AWERB Committee and the UK Home Office.

## Author Contributions

SS: conceptualization, validation, and project administration. SP, SR, and SS: methodology. AS, AI, RP, SR, and SS: formal analysis. AS, CM, AI, SN, RP, EF, PS, and SS: investigation. NR, SH, JG, and SS: resources. AI, SR, and SS: data curation. AS, AI, and SS: writing – original draft. AS, AI, NR, SP, SH, and SS: writing – review and editing. AS, AI, JS-A, and SS: visualization. NR, SP, SH, RC, and SS: supervision. NR, SH, JG, and SS: funding acquisition. All authors contributed to the article and approved the submitted version.

## Conflict of Interest

The authors declare that the research was conducted in the absence of any commercial or financial relationships that could be construed as a potential conflict of interest.
